# Targeting a distinct binding pocket in the pregnane X receptor with natural agonist TRLW-2 ameliorates murine ulcerative colitis

**DOI:** 10.3389/fphar.2025.1726597

**Published:** 2025-12-11

**Authors:** Shangrui Rao, Yi Mei, Lingyan Shi, Hongzheng Li, Minyu Zhou, Ziyu Meng, Zhijie Zhao, Shaotang Li, Weijian Sun, Qiang Tian

**Affiliations:** 1 Department of Colorectal and Anal Surgery, The First Affiliated Hospital of Wenzhou Medical University, Wenzhou, China; 2 Department of Oncology, Nanjing Drum Tower Hospital and Group’s Suqian Hospital, Affiliated Hospital of Medical School, Nanjing University, Nanjing, China; 3 College of Life Science and Health, Wuhan University of Science and Technology, Wuhan, China; 4 School of Basic Medical Sciences, Army Medical University, Chongqing, China

**Keywords:** ulcerative colitis, pregnane X receptor, distinct binding pocket, virtual screening, TRLW-2, cell proliferation

## Abstract

**Background:**

The therapeutic development of pregnane X receptor (PXR) agonists for ulcerative colitis (UC) is hindered by the poor selectivity of the canonical ligand-binding pocket. This study aimed to identify a novel, selectivity-enhancing binding site on PXR and a corresponding natural ligand for UC treatment.

**Methods:**

A distinct binding pocket (Pocket 1–5) within the PXR ligand-binding domain was identified using a multi-algorithm computational approach (SiteMap, Fpocket, Prank, CASTpFold). Structure-based virtual screening of 6,058 natural compounds from a traditional Chinese medicine library was performed via Glide docking (High-Throughput Virtual Screening/Standard Precision/Extra Precision modes, HTVS/SP/XP), with binding affinities refined by molecular mechanics/generalized Born surface area (MM/GBSA). The top candidate, TRLW-2 (catechin), was evaluated using luciferase reporter assays, quantitative real-time PCR (Qpcr), and functional assays (CCK-8, EdU, Annexin V-FITC/PI) in HEK293T and mouse colonic epithelial cells (MCECs). *In vivo* efficacy was assessed in a DSS-induced murine colitis model.

**Results:**

TRLW-2 exhibited high affinity for pocket 1-5, forming key hydrogen bonds with residues including Ser350, Asp352, Asp363, Thr398, and Arg401, which was validated by molecular dynamics simulations (MD) and site-directed mutagenesis. Functionally, TRLW-2 acted as a potent PXR agonist, significantly upregulating detoxifying enzymes (such as Cyp2b10, Cyp3a11 and Ugt1a1) and proliferation markers (PCNA, CDK1, Cyclin B1, and Ki67) *in vitro*. It promoted epithelial cell proliferation and inhibited apoptosis in MCECs. In DSS-induced colitis mice, TRLW-2 treatment significantly attenuated weight loss, reduced disease activity index DAI) and colonic mucosa damage index (CMDI) scores, ameliorated colon shortening, and improved histopathology.

**Conclusion:**

This study identified pocket 1–5 as a selectivity-enhancing site on PXR. The natural product TRLW-2, discovered via virtual screening, potently engages this pocket and demonstrates robust anti-inflammatory and mucosal healing effects, validating a promising strategy for developing precision therapeutics for UC.

## Graphical Abstract

**Figure:**
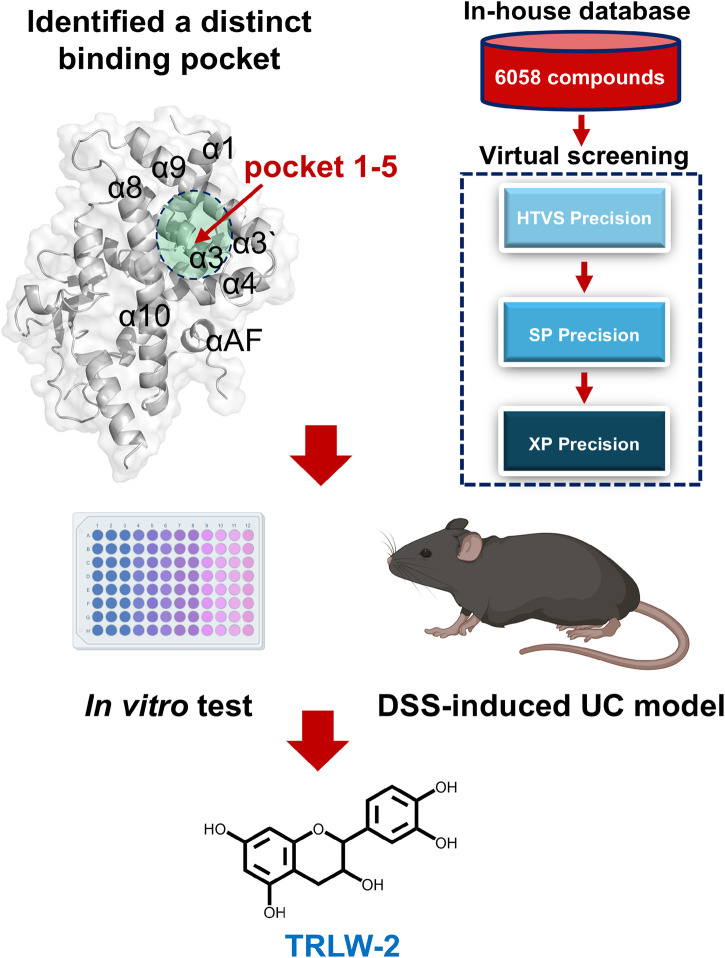

## 1 Introduction

Inflammatory bowel disease (IBD), encompassing Crohn’s disease (CD) and ulcerative colitis (UC), is a chronic and relapsing inflammatory disorder of the gastrointestinal tract, with a rapidly increasing global incidence, particularly in newly industrialized regions such as China (Banerjee et al., 2020; Agrawal et al., 2022; Ananthakrishnan et al., 2022). This growth, paralleling economic development, underscores the pressing need for enhanced healthcare infrastructure and preventive strategies to manage the escalating disease burden (Burisch et al., 2023). The pathogenesis of UC involves dysregulated immune responses, genetic susceptibility, gut microbiota dysbiosis, and environmental triggers, leading to sustained activation of pro-inflammatory pathways such as NF-κB and compromised epithelial barrier function (Liu et al., 2021; Gilliland et al., 2024). Current therapeutic strategies, including aminosalicylates, corticosteroids, immunomodulators, and biological agents such as anti-TNFα and IL-12/23 inhibitors, often fall short due to limited efficacy, significant side effects, and loss of response over time, with approximately one-third of patients exhibiting primary non-response and half eventually losing response (Agrawal et al., 2021; Baumgart and Le Berre, 2021; Rogler et al., 2021). These limitations highlight an urgent need for novel therapeutic agents that can more precisely target underlying inflammatory mechanisms with improved safety profiles, making the development of innovative drugs targeting emerging molecular pathways a critical priority in UC research.

The pregnane X receptor (PXR), a ligand-activated nuclear receptor highly expressed in the intestinal epithelial cells (IECs), serves as a critical xenobiotic sensor and regulator of mucosal homeostasis (Dvořák et al., 2020). It orchestrates the expression of detoxifying enzymes and transporters to facilitate xenobiotic metabolism and barrier protection against toxic luminal compounds (Feng et al., 2025). Beyond detoxification, PXR activation exerts potent anti-inflammatory effects by antagonizing NF-κB signaling, thereby suppressing pro-inflammatory cytokine production and enhancing epithelial integrity (Beuers et al., 2023; Zhang et al., 2023). Importantly, PXR interacts with gut microbiota-derived metabolites to modulate microbial composition, strengthen epithelial barrier function, and promote mucosal healing (Guan et al., 2022; Thibaut and Bindels, 2022). Dysregulation of PXR signaling is implicated in intestinal inflammation, highlighting its therapeutic potential for inflammatory bowel disease.

Several PXR agonists, such as rifaximin, have demonstrated potential in UC therapy by modulating inflammatory pathways and enhancing epithelial barrier integrity (Little et al., 2022; Carbonnel et al., 2025). However, many existing agonists suffer from non-specificity, off-target effects, or limited efficacy due to their additional biological activities and lack of selectivity for PXR over other nuclear receptors, which can lead to unintended systemic consequences or reduced therapeutic window (Brewer and Chen, 2016). For instance, rifampicin can upregulate cytochrome P450 enzymes, leading to the generation of hepatotoxic reactive metabolites from co-administered drugs like acetaminophen (Albrecht et al., 2019). Moreover, certain agonists exhibit species-specific activation profiles and poor pharmacokinetics, further constraining their clinical utility. These limitations underscore the necessity for *de novo* screening campaigns aimed at identifying novel, highly selective PXR ligands that leverage unique structural features of PXR’s flexible binding pocket to achieve enhanced efficacy and reduced adverse effects in UC treatment. The canonical ligand-binding pocket (LBP) of PXR is notoriously large and flexible, accommodating a wide array of chemically distinct ligands (Beuers et al., 2023). This promiscuity, however, comes at the cost of selectivity, often leading to unintended activation of off-target pathways. While this canonical pocket has been the primary focus for agonist development, its inherent flexibility presents a major challenge for designing selective drugs. Our hypothesis was that targeting a distinct, potentially more constrained binding site on PXR, distinct from the canonical LBP, could circumvent these limitations by enabling more specific ligand-receptor interactions.

The exploration of natural products for novel PXR agonists represents a highly promising and innovative strategy in UC drug discovery, owing to their structural diversity, high selectivity, and favorable safety profiles compared to synthetic compounds (Hiebl et al., 2018; Fox Ramos et al., 2019). Numerous phytochemicals, such as furanodienone (Wang X. et al., 2025), hyperforin (El Hamdaoui et al., 2022), and alpinetin (Yu et al., 2020), have demonstrated potent PXR activation, which modulates xenobiotic metabolism, suppresses NF-κB-mediated inflammation, and enhances epithelial barrier integrity. Importantly, many natural ligands exhibit tissue-specific activity, particularly in the colon, thereby minimizing systemic side effects (Zhao et al., 2020). Consequently, leveraging natural products not only aligns with a growing emphasis on holistic and complementary medicine but also accelerates the identification of lead compounds with optimized bioavailability and therapeutic potential for UC treatment. However, current research on natural product-derived PXR agonists reveals several critical limitations that hinder their therapeutic potential. A primary concern is their low ligand selectivity, as the large and flexible PXR binding pocket accommodates diverse structures but often results in off-target effects and unintended activation of other nuclear receptors, reducing therapeutic specificity (Zhang et al., 2020; Lin et al., 2023). Another major issue is the risk of adverse drug interactions. By potently activating PXR, these compounds can upregulate drug-metabolizing enzymes and transporters, thereby accelerating the metabolism of co-administered pharmaceuticals and potentially leading to toxicity or reduced drug efficacy (Gee et al., 2024). Consequently, there is a pressing need to screen for novel natural products that function as selective PXR modulators with improved safety profiles and minimized drug-interaction potential.

In this context, catechin, a naturally occurring flavan-3-ol abundant in green tea and various medicinal plants, presents a compelling candidate. It has a long history of use in Chinese traditional medicine and is widely recognized for its potent anti-inflammatory and antioxidant properties, which have been demonstrated in various disease models, including metabolic, arthritic, and neuroinflammatory disorders (de Araújo et al., 2021; Pyrzynska, 2025). These established biological activities align closely with the key pathological features of UC, such as chronic inflammation and oxidative stress. Furthermore, its favorable safety profile makes it an attractive starting point for drug development. Therefore, when our virtual screening identified catechin (designated as TRLW-2) as a high-affinity ligand for the distinct pocket 1–5, its selection for further investigation was strongly supported not only by its superior docking metrics but also by its promising and relevant pharmacological background.

We hypothesized that targeting a distinct selectivity-enhancing pocket on PXR with a natural ligand could overcome the limitations of current agonists. A multi-algorithm computational approach was employed to identify a distinct binding site (pocket 1–5), followed by structure-based virtual screening of a traditional Chinese medicine natural compound library to discover high-affinity ligands. This strategy aims to develop selective PXR modulators that effectively ameliorate intestinal inflammation while minimizing off-target effects and adverse drug interactions, offering a novel therapeutic avenue for UC.

## 2 Materials and methods

### 2.1 Chemicals, reagents and plasmids

Commercial chemicals and reagents, including catechin (HY-N0898, MCE, United States), aposcopolamine (HY-N8728, MCE, United States), an EdU Cell Proliferation Kit with AF594 (C0078S, Beyotime, China), a dual-luciferase reporter assay kit (DL101-01, Vazyme, China), an AceQ qPCR SYBR Green Master Mix kit (Q111-02, Vazyme, China), and a HiScript III RT SuperMix kit (R323-01, Vazyme, China) were purchased. XREM-luc (E4121) and pRL (E2261) plasmids were purchased from Promega. The cDNA sequence of PXR was cloned into the pLenti6/v5 lentiviral expression vector using the homologous recombination method.

### 2.2 Animals and treatment

A total of 18 male C57BL/6J mice (18–20 g) were purchased from Zhejiang Weitong Lihua Experimental Animal Technology Co., Ltd. (Zhejiang, China) and housed under specific pathogen-free (SPF) conditions at the Wenzhou Institute, University of Chinese Academy of Sciences. After 1 week of acclimatization, the mice were randomly assigned to three groups (n = 6 per group) based on body weight: a control group, a model group, and a treatment group. Colitis was induced in the model and treatment groups by administering 3% (w/v) DSS (MW 36,000–50,000, Meilunbio, China) dissolved in drinking water for 7 consecutive days. The treatment group received a daily oral gavage of TRLW-2 (10 mg/kg) concurrently with DSS administration. Body weight, stool consistency, and fecal occult blood were monitored daily. On day 15, colon tissues were collected after anesthesia with tribromoethanol (200 mg/kg), and serum samples were obtained by centrifugation (12,000 rpm, 10 min, 4 °C) for subsequent analysis. All animal experiments and procedures were conducted in strict accordance with the guidelines for the care and use of laboratory animals and were formally approved by the Animal Ethics Committee of the Wenzhou Institute, University of Chinese Academy of Sciences (Approval No. WIUCAS25092501).

### 2.3 DAI and CMDI

The DAI was calculated as the combined score of weight loss, stool consistency, and fecal blood, as previously described (Kou et al., 2025). The CMDI was evaluated based on colon tissue histopathology assessing epithelial damage, crypt destruction, and inflammatory cell infiltration.

### 2.4 Histopathological analysis

Colon tissue samples were collected on day 15, fixed in 4% paraformaldehyde for 24 h, and embedded in paraffin. Sections of 4 μm thickness were prepared and stained with hematoxylin and eosin (H&E) according to standard protocols. Images were captured using a light microscope (Nikon Eclipse Ci-L, Japan) equipped with a digital camera.

### 2.5 The prediction of binding pockets

The potential pockets of PXR (PDB: 9FZJ) were predicted using the Fpocket (https://fpocket.sourceforge.net/), CASTpFold (https://cfold.bme.uic.edu/castpfold/), Prank (https://prankweb.cz/), and SiteMap plugin module of Maestro (version 2021, Schrodinger, United States). Default parameters were used for all calculations. The potential binding pockets in PXR were visualized using PyMOL 3.0 (Schrodinger, United States).

### 2.6 In-house library preparation

The five classical Chinese medical texts including Prescriptions of the Bureau of Taiping People’s Welfare Pharmacy, Treatise on Cold Pathogenic Diseases, Treatise on the Spleen and Stomach, Synopsis of the Golden Chamber, and Effective Prescriptions for Universal Relief were systematically reviewed to identify traditional formulas used for treating gastrointestinal disorders. Representative formulations included Dahuang Gancao Decoction, Huangqi Jiangzhong Decoction, Banxia Xiexin Decoction, Xiaojianzhong Decoction, Sijunzi Decoction, Lizhong Pill, Baohe Pill, and Jishu Pill, among others. From these formulas, 128 herbal medicines were identified, such as *Bupleurum Chinense* DC., *Paeonia obovata* var. *glabra* Makino., *Atractylodes macrocephala* Koidz., *Pinellia ternata* (Thunb.) Makino, G*lycyrrhiza uralensis* Fisch. Ex DC., *Panax ginseng* C. A. Mey., *Dolomiaea souliei* (Franch.) C. Shih, *etc*. The natural small molecule compounds present in these herbs were then annotated using three online databases: the Traditional Chinese Medicine Systems Pharmacology Database and Analysis Platform (TCMSP, http://tcmspw.com/tcmsp.php), the Traditional Chinese Medicine Integrated Database (TCMID, http://www.megabionet.org/tcmid/), and the Chemical Database of Shanghai Institute of Organic Chemistry (https://organchem.csdb.cn). A total of 6,058 compounds were retrieved and compiled into an in-house library for further analysis.

### 2.7 Molecular docking and binding energy calculation

Molecular docking was performed using the Glide module in Schrodinger Maestro. The protein structure was prepared and optimized, and a docking grid was generated around the binding site (-pocket 1–5). A hierarchical screening strategy was employed, involving HTVS followed by SP and XP docking modes to efficiently identify high-affinity ligands with accurate binding poses. To more accurately evaluate the binding affinities of the docking hits, we performed binding free energy calculations using the MM/GBSA method via the Prime module on the poses generated from Glide XP docking. This method is widely recognized for providing a more rigorous estimate of binding energy compared to docking scores alone, as it incorporates solvation and entropy effects. The selection of final candidates was based on a consensus scoring strategy that combined the Glide Score from docking and the calculated ΔGbind from MM/GBSA. This was complemented by a detailed visual inspection of the ligand-protein interaction geometries using PyMOL 3.0, focusing on the stability and quality of the hydrogen-bonding networks with pharmacophorically important residues, to ensure plausible binding modes.

### 2.8 Transcriptional activity assay of PXR

The transcriptional activity of human pregnane X receptor (hPXR) was evaluated using a dual-luciferase reporter gene assay, as previously described with modifications (Huang et al., 2022). Briefly, 293T cells were seeded in 48-well plates at a density of 2 × 10^5^ cells per well and cultured for 24 h. The cells were then co-transfected with 200 ng of the reporter plasmid XREM-Luc, 100 ng of plasmid plenti6/v5-hPXR, and 5 ng of the internal control plasmid pRL using Opti-MEM medium and Lipofectamine 2000 (11668030, Invitrogen, United States) transfection reagent according to the manufacturer’s instructions. After 8 h of transfection, the medium was replaced with fresh culture medium containing the test compounds at the indicated concentrations. The cells were further incubated for 24 h. Luciferase activity was measured using the dual-luciferase reporter assay kit (DL101-01, Vazyme, China). The relative PXR transcriptional activity was expressed as the ratio of firefly to renilla lucuriferase activity.

### 2.9 CCK-8 assay

MCEC cells were seeded into 96-well plates at a density of 5 × 10^3^ cells per well in 100 μL of complete medium and pre-cultured for 24 h under standard conditions (37 °C, 5% CO_2_) to facilitate adhesion. After incubation, the cells were treated with various concentrations of test compounds and incubated for an additional 24 h. Following treatment, 10 μL of CCK-8 reagent (A311-01, Vazyme, China) was added to each well. The plate was then incubated at 37 °C for 4 h to allow for formazan formation. After incubation, the absorbance of each well was measured at 450 nm using a microplate reader.

### 2.10 Cell proliferation assay using EdU labeling

Cell proliferation was assessed using the BeyoClick EdU-594 Cell Proliferation Kit (C0078S, Beyotime, China) according to the manufacturer’s instructions. In brief, cells were incubated with 10 μM EdU for 4 h under standard culture conditions to allow incorporation of EdU into newly synthesized DNA. After labeling, cells were fixed with 4% paraformaldehyde, permeabilized with 0.3% Triton X-100, and incubated with the click reaction mixture containing alexa fluor 594 azide for 30 min at room temperature in the dark. The stained cells were visualized under a fluorescence microscope (BX53, Olympus, Japan). EdU-positive cells exhibited red fluorescence, indicating proliferating cells, while all nuclei were identified by blue fluorescence. The percentage of EdU-positive cells was calculated to evaluate cell proliferation activity.

### 2.11 Apoptosis detection via Annexin V-FITC/PI staining

Apoptosis was quantified by flow cytometry using an Annexin V-FITC/PI staining kit (A211-01, Vazyme, China). Briefly, both adherent and suspension cells were harvested, washed with cold PBS, and resuspended in 100 µL of 1× Binding Buffer. Then, 5 µL of Annexin V-FITC and 5 µL of PI staining solution were added, followed by incubation in the dark at room temperature for 10 min. After adding 400 µL of Binding Buffer, samples were analyzed immediately on a flow cytometer with 488 nm excitation. FITC fluorescence was detected in the FL1 channel and PI in the FL3 channel. Viable (Annexin V^−^/PI^−^), early apoptotic (Annexin V^+^/PI^−^), and late apoptotic/necrotic (Annexin V^+^/PI^+^) populations were distinguished based on fluorescence signals.

### 2.12 Cell culture

HEK293T and MCEC cell lines obtained from the Stem Cell Bank (Chinese Academy of Sciences) were cultured in Dulbecco’s Modified Eagle Medium (DMEM) supplemented with 10% FBS (BC-SE-FBS08, Sbjbio, China), 100 U/mL penicillin and 100 μg/mL streptomycin (60162ES76, Yesen, China). Cells were cultured in a humidified atmosphere with 5% CO_2_ at 37 °C.

### 2.13 Quantitative real-time PCR (q-PCR)

Q-PCR was performed to measure gene expression levels. Total RNA was extracted, reverse transcribed into cDNA, and amplified using a SYBR Green-based system with gene-specific primers. Reactions were carried out in triplicate on a real-time PCR instrument under standard cycling conditions. Relative expression was calculated using the 2^−ΔΔCT^ method with normalization to endogenous control genes, and melt curve analysis confirmed amplification specificity.

### 2.14 Statistical analysis

All results are expressed as mean ± SD. Statistical analysis was performed with two-tailed Student’s t-test or one-way ANOVA followed by Dunnett’s test or Tukey’s test or two-way ANOVA followed by Tukey’s multiple comparisons test using Prism 9.0 (GraphPad, United States). P ≤ 0.05, p ≤ 0.01, and p ≤ 0.001 were considered statistically significant and are indicated by *, **, and ***, respectively.

## 3 Results

### 3.1 Identification of small molecule-binding pockets in PXR

**FIGURE 1:**
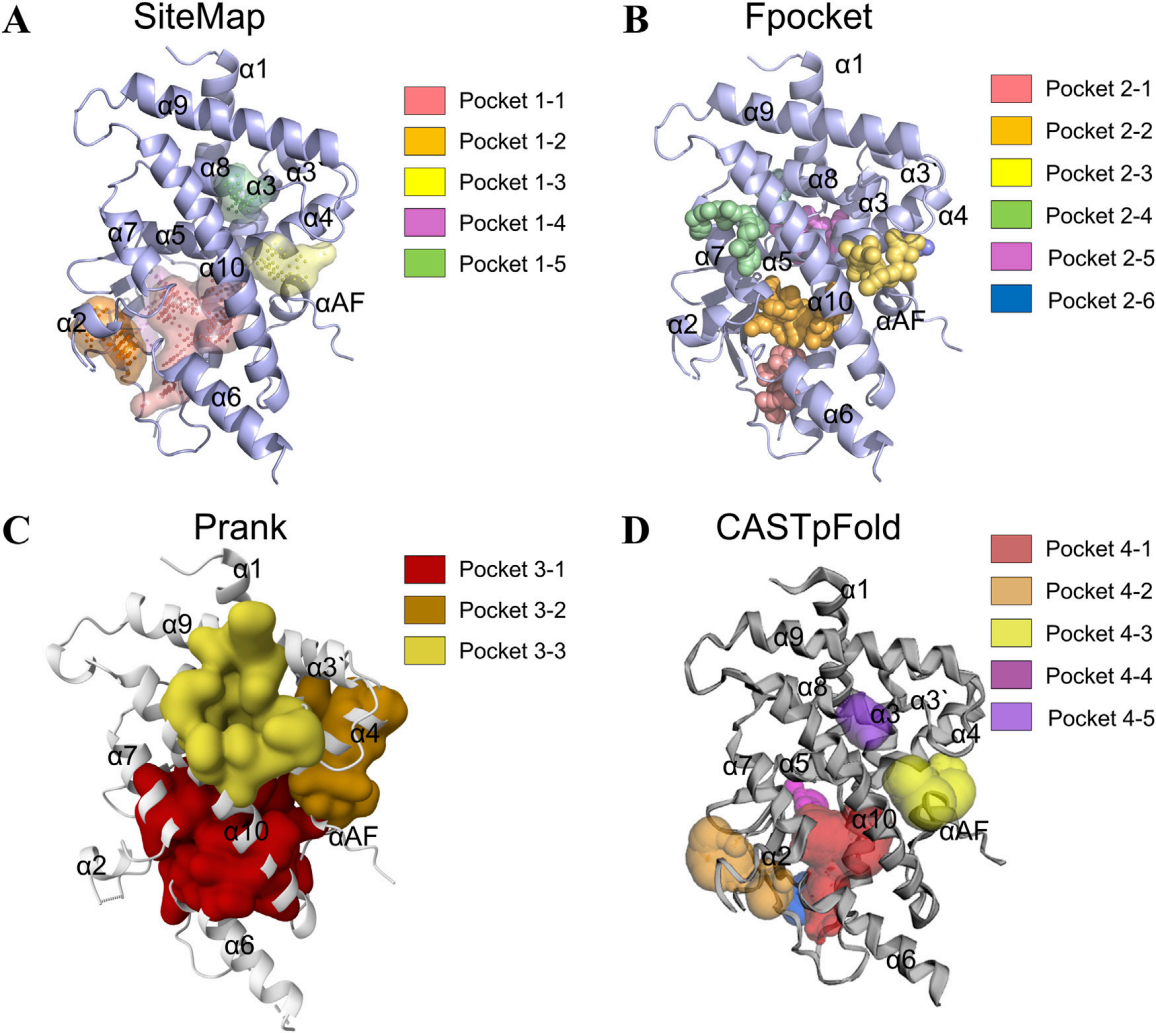
Identification of the binding pockets in PXR using different tools. **(A)** SiteMap prediction of five high-rank possible binding pockets in PXR. **(B)** Fpocket prediction of six high-rank possible binding pockets in PXR. **(C)** Prank prediction of three high-rank possible binding pockets in PXR. **(D)** CASTpFold prediction of five high-rank possible binding pockets in PXR.

To comprehensively identify potential ligand-binding sites on the PXR, we employed four distinct computational pocket detection algorithms: SiteMap, Fpocket, Prank, and CASTpFold. The comparative results visualized in Figure 1, demonstrate that while all methods successfully identified multiple putative binding pockets on the PXR protein surface, significant variability was observed in the number, precise location, and morphology of the predicted pockets. SiteMap analysis predicted a total of five distinct binding sites (Figure 1A). Notably, pocket 1-1 was localized in proximity to the α3, α7, and α10 helix of the protein’s canonical ligand-binding domain, occupying a significant volume. Fpocket identified the highest number of cavities, with six potential pockets (pocket 2-1 to pocket 2–6) (Figure 1B). The distribution of these pockets showed partial overlap with those predicted by other methods, yet also highlighted unique regions. In contrast, the Prank algorithm predicted three primary binding sites (pocket 3-1 to 3–3) (Figure 1C). This method yielded a more conservative estimate of pocket numbers compared to SiteMap and Fpocket. Similarly, CASTpFold predicted five binding pockets (pocket 4-1 to pocket 4–5) (Figure 1D). Pocket 4-1 was identified in a position analogous to pockets predicted by other methods in the central cavity of the ligand-binding domain.

### 3.2 Identification of a distinct binding pocket in PXR for selective agonist design

**FIGURE 2:**
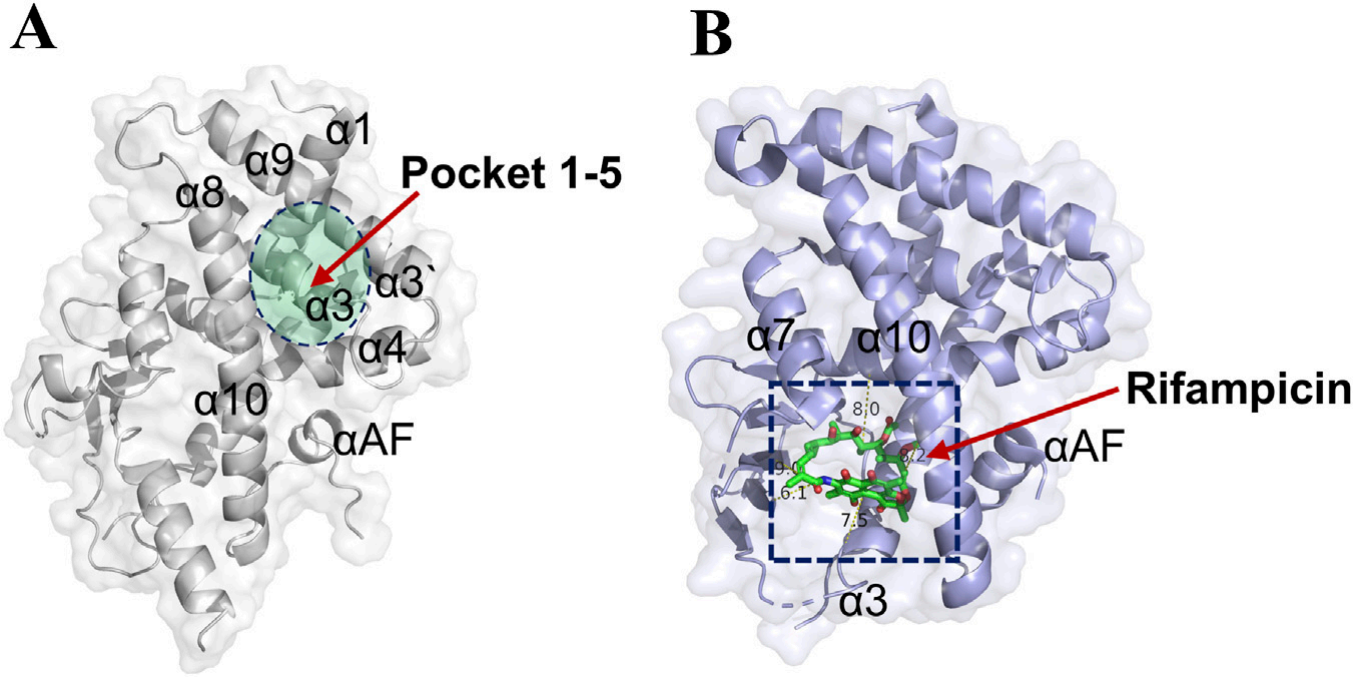
Pocket 1–5 was located in the α3, α4, α8, and α9 cavity. **(A)** The detail location information of pocket 1–5 (PDB: 9FZJ). **(B)** The crystal structure of Rifampicin-PXR complex (PDB:1SKX).

As shown in Figure 2A, a distinct binding pocket (pocket 1–5) was identified within the ligand-binding domain of the PXR. This pocket was situated within a cleft formed by several key alpha-helices, including α3, α4, α8, and α9. Its distinct structural features and relatively compact volume suggested potential for high-affinity and selective ligand interactions. In contrast, Figure 2B depicts the binding mode of the classic PXR agonist rifampicin, which occupies a larger and more flexible cavity within the receptor (Chrencik et al., 2005). Although rifampicin is a well-known agonist, its binding site involves similar helical elements but exhibits considerably less topological constraint, which may account for its promiscuous binding behavior and limited selectivity (Shehu et al., 2019). Further supporting this observation, Supplementary Figure S1 presents the binding modes of two additional PXR agonists: SR12813 and Furanodienone. While SR12813 binds a relatively confined sub-pocket, Furanodienone occupies a broader region, consistent with the notion that conventional agonists tend to exploit larger binding interfaces with considerable conformational flexibility (Wang X. et al., 2025). This binding promiscuity likely contributes to their reduced selectivity and increased potential for off-target effects.

The discovery of pocket 1-5 offers a promising alternative for designing more selective PXR modulators. Its well-defined architecture and unique spatial characteristics differentiate it from the known binding sites associated with typical agonists. This structural novelty may facilitate the development of ligands with improved specificity and reduced adverse effects, thereby addressing critical limitations of existing PXR-targeting compounds.

### 3.3 Screening of PXR agonists within the in-house chemical library

**FIGURE 3:**
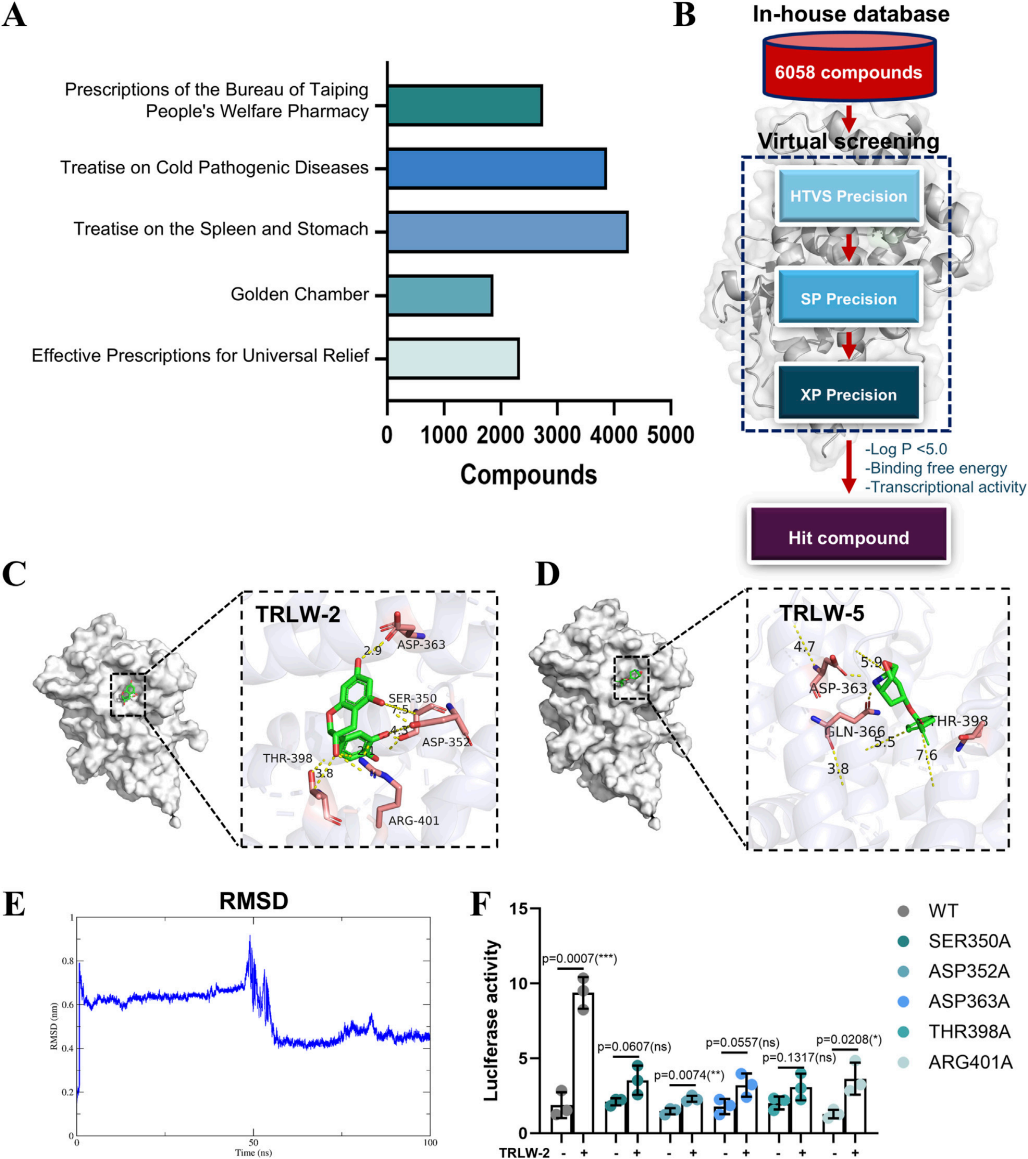
Virtual screening identified TRLW-2 binding in Pocket 1–5. **(A)** The compounds of five major TCM pharmacopoeias. **(B)** The workflow of structure-based virtual screening based on in-house natural compound library. **(C)** The docking model of TRLW-2 in the pocket 1–5 (PDB:1SKX). **(D)** The docking model of TRLW-5 in the pocket 1–5 (PDB:1SKX). **(E)** Root-mean-square deviation (RMSD) of the protein backbone throughout the 100 ns molecular dynamics simulation. **(F)** Functional impact of point mutations on PXR activation by TRLW-2. Relative luciferase activity of wild-type (WT) PXR and the indicated point mutants in response to TRLW-2 treatment was shown.

The foundation of this study was a classical TCM formula with documented efficacy for UC. The distribution of these compounds across five major TCM pharmacopoeias that document similar formulations is summarized in Figure 3A. We constructed a comprehensive in-house database containing 6,058 compounds isolated from these TCM pharmacopoeias. Virtual screening was performed using molecular docking (HTVS/SP/XP modes) (Figure 3B). Based on favorable MM/GBSA-derived binding free energy (ΔGbind) and drug-like Log P values, the top 10 compounds were selected as candidates (Supplementary Figure S2). The docking results (Table 1) corroborated the findings from the prior MM/GBSA analysis, with TRLW-2 confirming its status as the top-ranking candidate by exhibiting the most favorable docking score of −9.819. This was followed by TRLW-5 (−8.52) and TRLW-3 (−7.514), collectively identifying them as the most promising leads with the strongest predicted interactions with the target protein. Molecular docking analysis revealed that TRLW-2 occupies the pocket 1-5 of LXR, forming hydrogen bonds with Ser350, Asp352, Thr398, Arg401, and Asp363 (Figure 3C). TRLW-5 formed hydrogen-bonding interactions with Gln366, Asp363, and Thr398 with PXR (Figure 3D). These candidates exhibit strong predicted binding interactions with PXR, highlighting their potential for further experimental validation and drug development.

**TABLE 1:** The docking score of the top 10 compounds.

No.	Compound	Docking score
1	TRLW-1	−5.984
2	TRLW-2	−9.819
3	TRLW-3	−7.514
4	TRLW-4	−6.518
5	TRLW-5	−8.52
6	TRLW-6	−6.975
7	TRLW-7	−6.808
8	TRLW-8	−6.144
9	TRLW-9	−6.084
10	TRLW-10	−6.729

To further validate the stability of the predicted TRLW-2-PXR complex, we performed molecular dynamics (MD) simulations. The root-mean-square deviation (RMSD) of the protein backbone stabilized after an initial equilibration phase, maintaining a stable plateau with minimal fluctuations for the duration of the 100 ns simulation (Figure 3E), indicating that the complex reached a stable conformational state. Complementing this, the radius of gyration (Rg) analysis of the protein backbone showed consistent values throughout the simulation (Supplementary Figure S2B), suggesting no major unfolding and overall structural compactness. These results collectively demonstrate the stability and structural integrity of the TRLW-2-PXR complex.

We next sought experimental validation of the binding mode through site-directed mutagenesis of key residues within the ligand-binding pocket. Consistent with the docking predictions, the PXR agonist activity of TRLW-2 was almost completely abolished in the Ser350A, Asp352A, Asp363A, Thr398A, and Arg401A mutants (Figure 3F), providing strong functional evidence that these residues are critical for TRLW-2 binding and PXR activation.

Our integrated computational strategy addresses this gap by systematically identifying high-affinity candidates from a vast chemical repertoire. By prioritizing candidates like TRLW-2 and TRLW-5, we provide a rational framework for elucidating the therapeutic basis of TCM formulations in UC.

### 3.4 TRLW-2 functions as a potent PXR agonist with enhanced transcriptional activity *in vitro*

**FIGURE 4:**
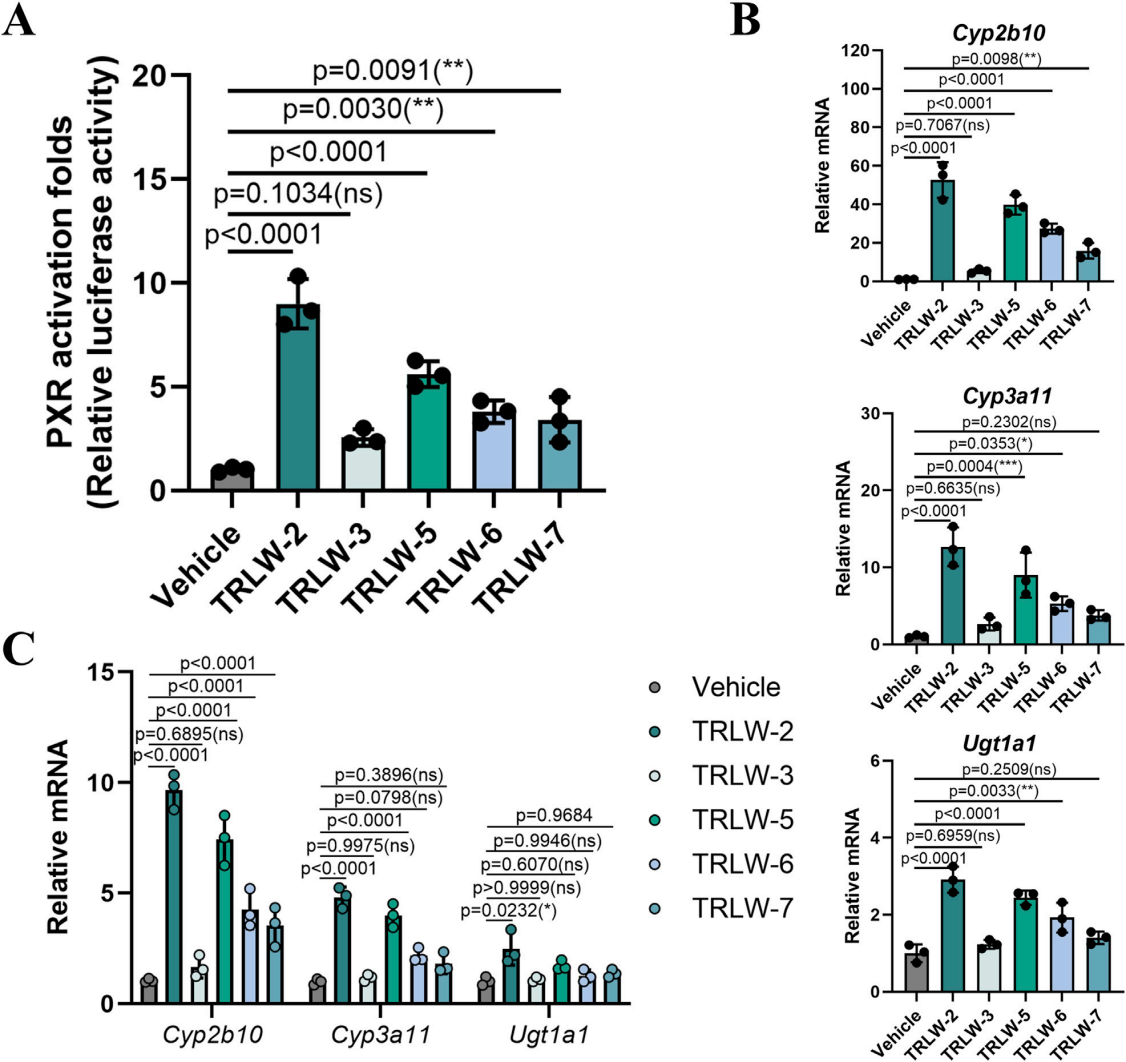
The functional characterization of the five candidate compounds. **(A)** Luciferase reporter assay evaluating the transcriptional activity of PXR with the five candidate compounds in 293T cells. **(B)** The downstream gene of PXR under the treatment of the five candidate compounds in 293T cells. **(C)** The downstream gene of PXR under the treatment of the five candidate compounds in MCEC cells. Data were expressed as the mean ± SD. Data are analyzed by one-way analysis of variance (ANOVA) followed by Dunnett’s test. *p < 0.05, **p < 0.01, ***p < 0.001; ns, not significant.

Based on the binding free energy calculations obtained from the MM/GBSA analysis, five candidate compounds including TRLW-2, TRLW-3, TRLW-5, TRLW-6, and TRLW-7 were selected for further pharmacological evaluation *in vitro*. Cytotoxicity assessment demonstrated that all compounds exhibited minimal cytotoxicity at the tested concentrations in MCEC cells (Supplementary Figure S3). Quantitative analysis determined the IC_50_ values for TRLW-2 and TRLW-5 to be > 200 μM, significantly higher than the effective concentration used in functional assays. Consequently, a uniform concentration of 200 nM was employed for all subsequent transcriptional activity assays. The functional characterization of the five candidate compounds identified TRLW-2 and TRLW-5 as potent activators of PXR-mediated transcription in a reporter assay performed in 293T cells (Figure 4A). Subsequent evaluation of downstream gene expression revealed that both compounds significantly upregulated the classic PXR target genes *Cyp2b10*, *Cyp3a11*, and *Ugt1a1,* resulting in remarkable approximately 58.2-fold (p < 0.0001),13.5-fold (p < 0.0001), and 2.8-fold increases, respectively, compared to the vehicle (Figure 4B). Crucially, this induction was consistently recapitulated MCEC cells, with TRLW-2 demonstrating particularly robust and reliable efficacy (Figure 4C).

The transition from virtual screening to functional validation is a pivotal milestone in drug discovery. Our results firmly establish TRLW-2 as a highly promising PXR agonist. Its ability to potently activate PXR and induce a suite of detoxification and anti-inflammatory genes. The potent induction of *Ugt1a1* is of particular therapeutic interest, as this enzyme facilitates the inactivation of luminal toxins and helps mitigate intestinal inflammation by enhancing phase II detoxification processes (Gotoh-Saito et al., 2025). The upregulation of *Cyp2b10* and *Cyp3a11* is equally noteworthy, as these cytochrome P450 enzymes are instrumental in the metabolism of a wide range of exogenous and endogenous compounds, including toxins and bile acids (Zhang et al., 2018). Although TRLW-5 also exhibited strong activation in the initial reporter assay, its performance in inducing downstream genes, especially *Ugt1a1*, was less consistent than that of TRLW-2. This divergence suggests that despite a shared origin from the same virtual screen targeting a distinct pocket, TRLW-2 may possess superior binding properties, metabolic stability, or selectivity, making it a more suitable candidate for further development. The absence of cytotoxicity at the effective concentration further underscores its potential as a safe and effective therapeutic agent.

### 3.5 TRLW-2 promoted the proliferation of colorectal epithelial cell

**FIGURE 5:**
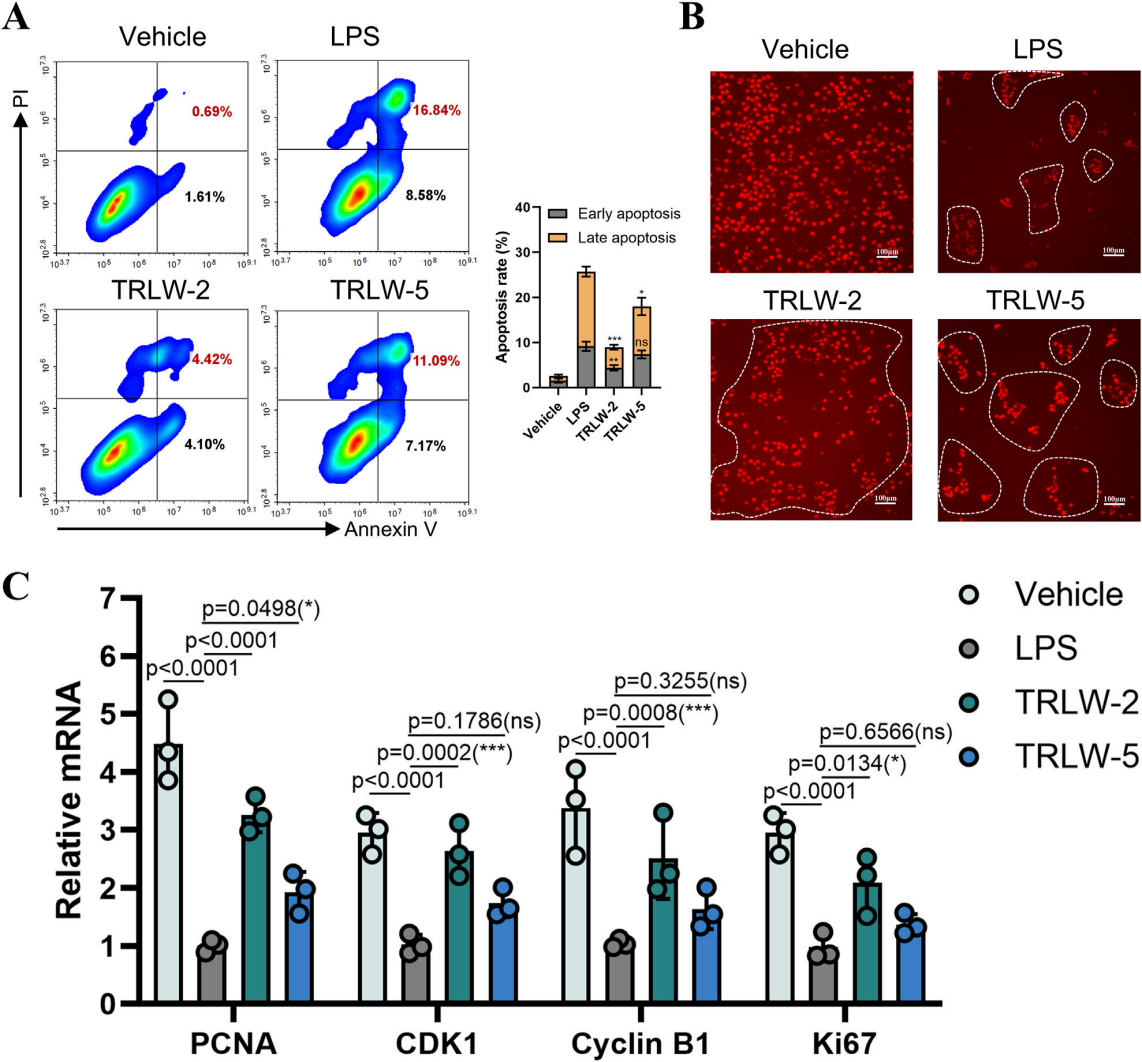
TRLW-2 promoted the proliferation of MCEC cells *in vitro*. **(A)** The apoptosis detection via Annexin V-FITC/PI staining with TRLW-2 and TRLW-5. **(B)** Representative images of EdU staining (red) showing proliferating cells in the colonic epithelium, the scale bar represents 100 μm. **(C)** The expression of the proliferation-related markers under the treatment of TRLW-2 and TRLW-5. Data were expressed as the mean ± SD. Data are analyzed by one-way analysis of variance (ANOVA) followed by Dunnett’s test. *p < 0.05, **p < 0.01, ***p < 0.001; ns, not significant.

Evaluation of TRLW-2 and TRLW-5 in MCEC demonstrated substantial beneficial effects on cellular viability and proliferation. Apoptosis assays revealed that both compounds significantly attenuated LPS-induced programmed cell death, with flow cytometry indicating a notable reduction in the proportion of cells in both early and late apoptotic stages following treatment (Figure 5A). These anti-apoptotic effects were accompanied by robust pro-proliferative activity, as evidenced by a marked increase in EdU-positive cells upon exposure to either compound (Figure 5B). Further supporting these observations, gene expression analysis showed significant upregulation of proliferation-related markers including *PCNA*, *CDK1*, *Cyclin B1*, and *Ki67* (Figure 5C). Interestingly, while both compounds exhibited favorable activity profiles, TRLW-2 consistently induced stronger pro-proliferative and anti-apoptotic responses compared to TRLW-5. The ability of these two compounds to enhance epithelial proliferation and viability carries particularly important implications for UC therapeutics.

### 3.6 TRLW-2 ameliorated DSS-induced colitis in mice

**FIGURE 6:**
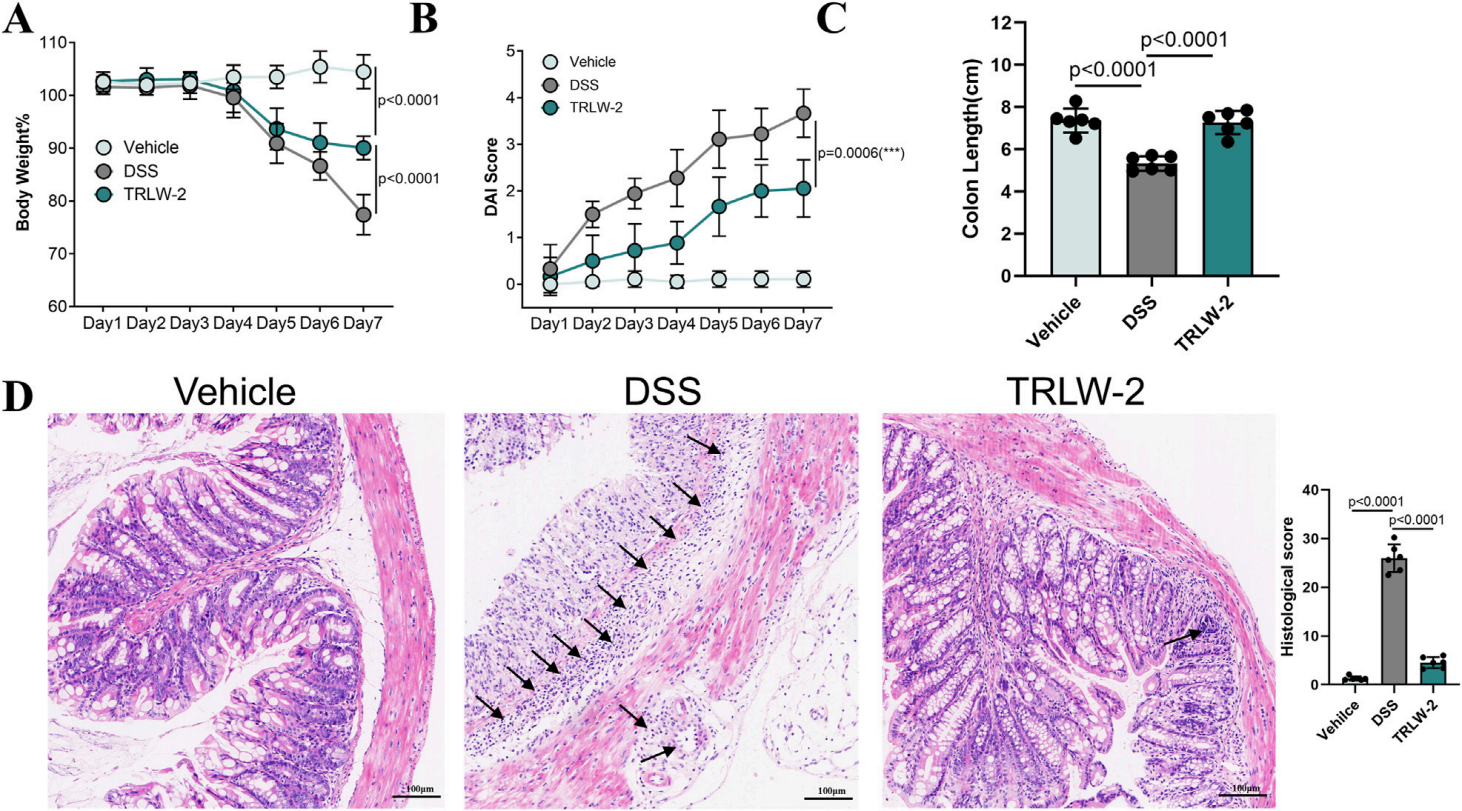
TRLW-2 ameliorated DSS-induced colitis in mice *in vivo*. **(A)** The body weight changes. **(B)** DAI score. **(C)** Changes in the length of colon tissues. **(D)** Representative H&E-stained colon sections. Black arrows indicate areas of significant inflammatory cell infiltration. Data were expressed as the mean ± SD. Data are analyzed by one-way analysis of variance (ANOVA) followed by Dunnett’s test. *p < 0.05, **p < 0.01, ***p < 0.001; ns, not significant.

The therapeutic potential of TRLW-2, a distinct PXR agonist identified through structure-based virtual screening targeting the newly discovered pocket 1–5, was rigorously evaluated in a DSS-induced murine model of colitis. The administration of TRLW-2 (10 mg/kg) significantly ameliorated the characteristic symptoms of colitis. Specifically, TRLW-2 treatment markedly attenuated DSS-induced body weight loss (Figure 6A), reduced the DAI score (Figure 6B), and prevented the shortening of colon length (Figure 6C) compared to the DSS-only group. Furthermore, analysis of supplementary data confirmed that TRLW-2 significantly improved the colon index (Supplementary Figure S4A) and reduced the CMDI score (Supplementary Figure S4B). To further investigate the anti-inflammatory mechanism of TRLW-2, we quantified the mRNA expression of key pro-inflammatory cytokines in colon tissues using q-PCR. As shown in Supplementary Figure S4C, treatment with TRLW-2 markedly attenuated this increase, resulting in significantly lower mRNA levels of all four cytokines (p < 0.0001). The suppression of these critical mediators of intestinal inflammation provides direct molecular evidence supporting the potent anti-inflammatory effects of TRLW-2 observed in our histological evaluations (Figure 6D).

The robust efficacy of TRLW-2 represents a critical validation of both the compound’s therapeutic potential *in vivo* and the innovative drug discovery strategy that identified it. This multifaceted efficacy likely stems from its potent activation of PXR-mediated protective responses, including the induction of detoxifying enzymes (*Cyp3a11*, *Ugt1a1*) and proliferative genes (*PCNA*, *Ki67*) as established in our previous *in vitro* studies. Most significantly, the outstanding performance of TRLW-2 validates the pioneering approach of targeting the distinct pocket 1-5 through virtual screening. Traditional PXR drug discovery has been hampered by the receptor’s large, promiscuous ligand-binding pocket, often resulting in non-selective compounds with off-target effects. Our strategy of targeting a more constrained, cryptic allosteric pocket has successfully yielded a ligand with exceptional *in vivo* efficacy and a favorable safety profile. These compelling results firmly establish TRLW-2 as a highly promising preclinical candidate for UC treatment *in vivo*, while simultaneously providing definitive proof-of-concept that pocket 1-5 represents a viable and innovative therapeutic target for developing next-generation PXR modulators with enhanced specificity and translational potential.

## 4 Discussion

The management of UC remains a significant clinical challenge due to the limitations of existing therapies, including 5-aminosalicylic acid, corticosteroids, and immunomodulators, which often exhibit suboptimal efficacy, high relapse rates, and systemic toxicity (Shen et al., 2022). Biological agents and JAK inhibitors, while offering advanced options, are constrained by primary non-response, safety concerns, and limited efficacy against extraintestinal manifestations (Honap et al., 2024). PXR agonists, such as rifampicin, have emerged as potential therapeutics for UC due to their role in regulating detoxification enzymes and bile acid metabolism. However, their clinical utility is hampered by promiscuous ligand binding and off-target effects largely attributable to the large, flexible, and poorly selective canonical ligand-binding pocket of PXR (Chrencik et al., 2005). Our study addresses these limitations by identifying a distinct cryptic pocket (pocket 1–5) within PXR and demonstrating that targeting this site with TRLW-2 confers potent anti-inflammatory and mucosal repair effects in UC models. A key distinction between pocket 1–5 and the canonical PXR binding site lay in their topology and implied function. The canonical pocket was characterized by its large volume and conformational flexibility, which allowed it to function as a generalized sensor for diverse xenobiotics but also contributed to low ligand selectivity and off-target effects (Chrencik et al., 2005; Shehu et al., 2019). In contrast, pocket 1–5 exhibited distinct spatial constraints and a compact volume, situated within a cleft formed by helices a3, a4, a8, and a9 (Figure 2A). This well-defined architecture enabled precise hydrogen-bonding interactions with residues such as Ser350, Asp352, Thr398, Arg401, and Asp363, as observed in the TRLW-2 docking model (Figure 3C). We proposed that this structural difference was fundamental to the enhanced selectivity observed with TRLW-2. The high topological specificity of pocket 1–5 likely reduced he probability of promiscuous binding, thereby minimizing off-target effects while promoting selective PXR activation. This contrasted with traditional agonists like rifampicin, which engaged the flexible canonical pocket in a manner that lacked similar geometric constraints, accounting for their limited specificity.

The existence of this structurally constrained pocket raised intriguing questions about its physiological role. We speculated that PXR might have evolved this secondary binding site to facilitate fine-tuned regulation by endogenous ligands or specific microbial metabolites. While the large, flexible canonical pocket allowed PXR to act as a broad sensor for xenobiotics, the more defined pocket 1–5 could serve as a selectivity filter or an allosteric regulatory site. This might have enabled a layered regulatory mechanism: the canonical pocket provided a coarse, generalized response to foreign compounds, whereas engagement of pocket 1–5 could modulate PXR activity in a more precise, tissue-specific, or pathway-selective manner, potentially in response to distinct internal signals. This suggested a previously unappreciated level of sophistication in the body’s regulation of detoxification and mucosal homeostasis, moving beyond a simple on/off switch to a more nuanced control system. This underscored the utility of pocket 1–5 for developing selective PXR agonists. Targeting this cryptic site might have stabilized PXR conformations that favored beneficial pathways while minimizing dysregulated metabolic effects.

It is noteworthy that the primary objective of this *in vivo* study was to provide a rigorous proof-of-concept for the therapeutic efficacy of engaging the novel pocket 1–5 with TRLW-2, rather than to conduct a direct comparative efficacy benchmark against established agonists like rifampicin. The classic agonists bind to the large, flexible canonical pocket, which is associated with promiscuous effects and limited selectivity. Consequently, their efficacy and potential side-effect profiles in the DSS model may not serve as an ideal benchmark for TRLW-2, which operates through a distinct, selectivity-enhancing mechanism. The canonical ligand-binding pocket of PXR is characterized by high conformational flexibility, allowing it to accommodate chemically diverse ligands but resulting in low selectivity and unintended activation of divergent pathways (Feng et al., 2025). This structural promiscuity explains the limited specificity of traditional PXR agonists like rifampicin, SR12813, and furanodienone, which occupy broad cavities with minimal topological constraints. In contrast, the newly identified pocket 1-5 exhibited distinct spatial constraints and a compact volume, which we hypothesize was central to achieving enhanced selectivity. The pocket 1-5 that situated within a cleft formed by helices α3, α4, α8, and α9 exhibited distinct spatial constraints and a compact volume (Figure 2A). Critically, our computational model predicted that this well-defined architecture enables precise hydrogen-bonding interactions with residues such as Ser350, Asp352, Thr398, Arg401, and Asp363 (Figure 3C). The functional importance of these predicted interactions was experimentally confirmed by our site-directed mutagenesis studies, which demonstrated that mutation of these key residues (S350A, D352A, etc.) significantly attenuated or abolished TRLW-2-induced PXR activation (Figure 3F). We therefore propose that this structural specificity, now supported by both computational and experimental evidence, was fundamental to the enhanced selectivity observed with TRLW-2. The high topological specificity of pocket 1-5 likely reduces off-target binding while promoting selective PXR activation, addressing a key drawback of conventional PXR-targeted agents. This underscores the utility of pocket 1-5 for developing selective PXR agonists. Targeting this cryptic site may stabilize PXR conformations that favor beneficial pathways while minimizing dysregulated metabolic effects. The promising preclinical profile of TRLW-2 suggested its potential as a candidate for further development. However, an important consideration for its future translational application, particularly within a precision medicine framework, was the impact of genetic variation. PXR exhibits polymorphisms that could significantly alter its expression and activity, thereby influencing individual responses to PXR-targeted therapies (Chen et al., 2019). While the current study demonstrated the efficacy of TRLW-2 against the standard reference receptor, future investigations were identified as a priority to evaluate its affinity and activation potential across common human PXR polymorphic variants. Such research was considered critical for determining whether TRLW-2 could have broad applicability or if its use might need to be tailored to specific patient genotypes, ultimately advancing its development towards truly personalized UC treatment.

UC is characterized by progressive epithelial damage, loss of barrier integrity, and impaired mucosal healing (Villablanca et al., 2022; Neurath et al., 2025). The intestinal epithelium serves as a critical physical and immunological barrier, and its regeneration is essential for maintaining homeostasis (Kudelka et al., 2020). PXR is highly expressed in colorectal epithelial cells, which plays a central role in preserving intestinal barrier function by modulating key processes such as epithelial repair, immune regulation, and microbial balance (Dvořák et al., 2020). Virtual screening prioritized TRLW-2 and TRLW-5 as high-affinity binders to the distinct pocket 1-5 of PXR. The observed upregulation of proliferation markers such as *PCNA*, *CDK1*, *Cyclin B1*, and *Ki67* suggests that TRLW-2 and TRLW-5 enhance mitotic activity and support epithelial repair mechanisms, directly countering aberrant epithelial turnover and ulceration in UC. Furthermore, the anti-apoptotic effects contribute to epithelial preservation under inflammatory stress, reducing barrier breakdown and subsequent antigen exposure that drives immune activation (Arnould et al., 2025). The superior pro-proliferative profile of TRLW-2, especially through the induction of genes central to cell cycle progression, positions it as a particularly promising candidate. Its efficacy in enhancing epithelial regeneration suggests potential not only in mitigating inflammation but also in restoring physiological barrier function, addressing a fundamental aspect of UC pathology. Additionally, PXR-driven metabolic pathways may detoxify luminal toxins and bile acids through *Cyp3a11*, *Ugt1a1*, further alleviating epithelial stress in UC (Yao et al., 2022). Its efficacy in enhancing epithelial regeneration suggests potential not only in mitigating inflammation but also in restoring physiological barrier function, thereby addressing a fundamental aspect of UC pathology.

TRLW-2’s selectivity against other nuclear receptors was an important therapeutic consideration. Although the canonical PXR ligand-binding pocket shares structural similarities with receptors such as CAR and FXR (Fiorucci et al., 2021), raising the possibility of off-target effects. Our data indicated that TRLW-2’s binding to the distinct pocket 1–5 likely enhanced its selectivity. The unique conformation of pocket 1–5 appeared less compatible with related receptors, potentially minimizing cross-reactivity commonly seen with traditional PXR agonists targeting the promiscuous canonical site. While further profiling was needed to fully confirm specificity across the nuclear receptor superfamily, the combined evidence from docking, MD simulations, and mutagenesis provided a strong structural and functional basis for its PXR-specific action, reducing the risk of off-target signaling.

The translation of TCMs into evidence-based therapies remains challenging due to the complexity of multi-component formulations and the unclear mechanisms of their active ingredients (Zhang et al., 2024; Li et al., 2025; Wang Z. et al., 2025). Herbs such as ginseng (Niu et al., 2024), bupleurum (Dong et al., 2019), and licoric (Xia et al., 2023) have historically been used for UC treatment, yet their specific bioactive compounds and molecular targets are poorly defined. Catechin (TRLW-2) is abundant in various dietary sources, particularly in green tea, which has been historically consumed and is recognized for its anti-inflammatory and antioxidant properties in traditional medicine (Kang et al., 2022; Feng et al., 2023). Notably, its efficacy has been extensively reported in arthritis (Goyal et al., 2023), metabolic and neuroinflammatory disorders (Lee et al., 2023), and chronic bacterial prostatitis (Chiavaroli et al., 2022). As a natural product, it generally exhibits favorable safety and tolerability profiles. Its relatively low molecular weight and structural characteristics contribute to acceptable bioavailability, although strategies to enhance its stability and absorption, such as nano-formulations as structural analogs (Jin et al., 2024), could be explored to further improve its pharmacokinetic properties for clinical application. Despite its broad anti-inflammatory effects and proven efficacy in diseases with overlapping pathophysiology, catechin remains underexplored in UC contexts. Focused research on bioavailability optimization, dose standardization, and UC-specific preclinical models could unlock its utility as a novel therapeutic agent for UC. This gap may stem from the fact that existing research has largely focused on mixed catechin preparations (such as green tea extracts) rather than in-depth mechanistic exploration of the single compound. Future investigations could focus on profiling the downstream transcriptional alterations of genes following PXR activation by the compound TRLW-2. Integrating these findings into a comprehensive molecular network would help decipher the precise mechanism through which it ameliorates UC.

While the current study demonstrates the robust efficacy of TRLW-2 in an acute colitis model, the long-term effects on intestinal health represent an important future direction. The favorable safety profile of catechin, documented in studies of chronic conditions such as arthritis and metabolic disorders where it was administered over extended periods, provides a positive indication for its potential sustained use (Goyal et al., 2023; Lee et al., 2023). Based on its mechanism of action, promoting epithelial repair and sustaining the expression of detoxifying enzymes, we hypothesized that TRLW-2 could contribute to long-term mucosal homeostasis and reduce relapse rates in UC. Future studies employing chronic or relapse-recurrence models of colitis will be essential to directly evaluate the long-term therapeutic benefits and durability of TRLW-2 treatment, a critical step in advancing its clinical translation.

## 5 Conclusion

This study successfully identified a distinct binding site (pocket 1–5) on PXR, providing a structural basis for developing agonists with enhanced selectivity. The natural compound TRLW-2 (catechin), discovered via virtual screening targeting this pocket, demonstrated potent PXR activation. It conferred significant protection against experimental colitis by inducing cytoprotective genes and promoting epithelial repair, without evident toxicity. The efficacy of TRLW-2 underscored the therapeutic promise of targeting pocket 1–5 and validated our computational strategy for repurposing natural products. Given its natural origin and the favorable safety profile observed in our study, TRLW-2 may represent a promising worthy of further investigation as a potential therapeutic agent for UC. Future studies should focus on optimizing its bioavailability and elucidating its full mechanistic network in PXR-mediated mucosal healing.

## Data Availability

The original contributions presented in the study are included in the article/Supplementary Material, further inquiries can be directed to the corresponding authors.

## Supplementary material

SUPPLEMENTARY FIGURE S1 The crystal structure of ligands and PXR. **(A)** The crystal structure of SR12813-PXR complex (PDB: 9FZJ). **(B)** The crystal structure of Furanodienone-PXR complex (PDB: 8R00).

SUPPLEMENTARY FIGURE S2 Computational analysis of PXR-ligand interactions and protein dynamics. **(A)** MM/GBSA analysis of the binding free energies of the top 10 compounds to PXR. **(B)** Radius of gyration (Rg) analysis of the PXR protein backbone during the 100 ns molecular dynamics simulation.

SUPPLEMENTARY FIGURE S3 The cytotoxicity assessment of the five candidate compounds in MCEC cells.

SUPPLEMENTARY FIGURE S4 The changes in inflammatory factors. **(A)** The changes in colon index. **(B)** The changes in CMDI score. **(C)** The mRNA expression of colon tissues. Data were expressed as the mean ± SD. Data are analyzed by one-way analysis of variance (ANOVA) followed by Dunnett’s test and two-way ANOVA followed by Tukey’s multiple comparisons test. *p < 0.05, **p < 0.01, ***p < 0.001; ns, not significant.

## Abbreviations

UC, Ulcerative Colitis; PXR, pregnane X receptor; IBD, Inflammatory bowel disease; TCM, traditional Chinese medicine; MCEC, mouse colonic epithelial cells; DAI, disease activity index; CMDI, colonic mucosa damage index; H&E, hematoxylin and eosin; Q-PCR, quantitative real-time PCR; MD, molecular dynamics; RMSD, root mean square distance/deviation; MM/GBSA, molecular mechanics generalized born surface area.
