# Supplementary figures and images for: Targeting a distinct binding pocket in the pregnane X receptor with natural agonist TRLW-2 ameliorates murine ulcerative colitis
Source: Front Pharmacol. 2025 Dec 11;16:1726597. doi: 10.3389/fphar.2025.1726597 (PMC12738937; doi:10.3389/fphar.2025.1726597)

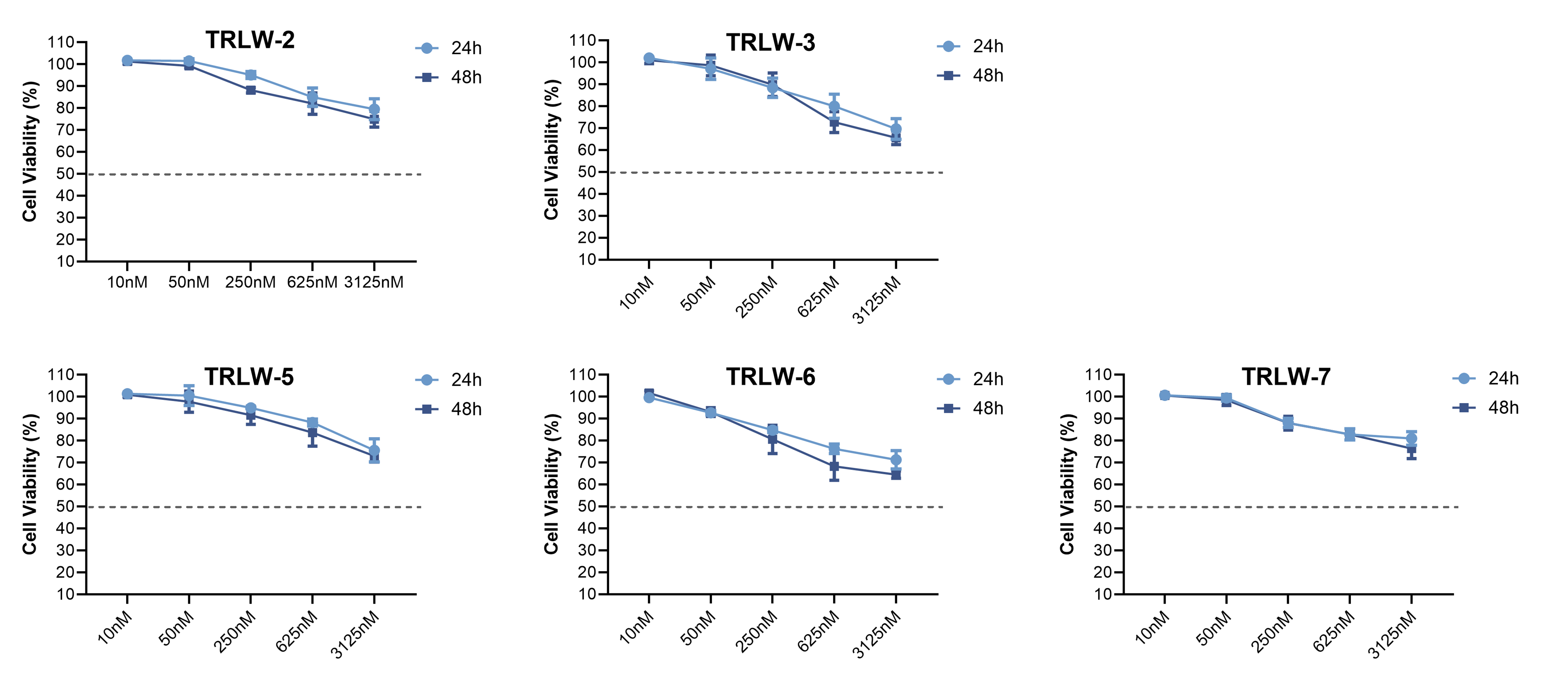

Supplement: Supplementary file 1 [file Image3.tif]

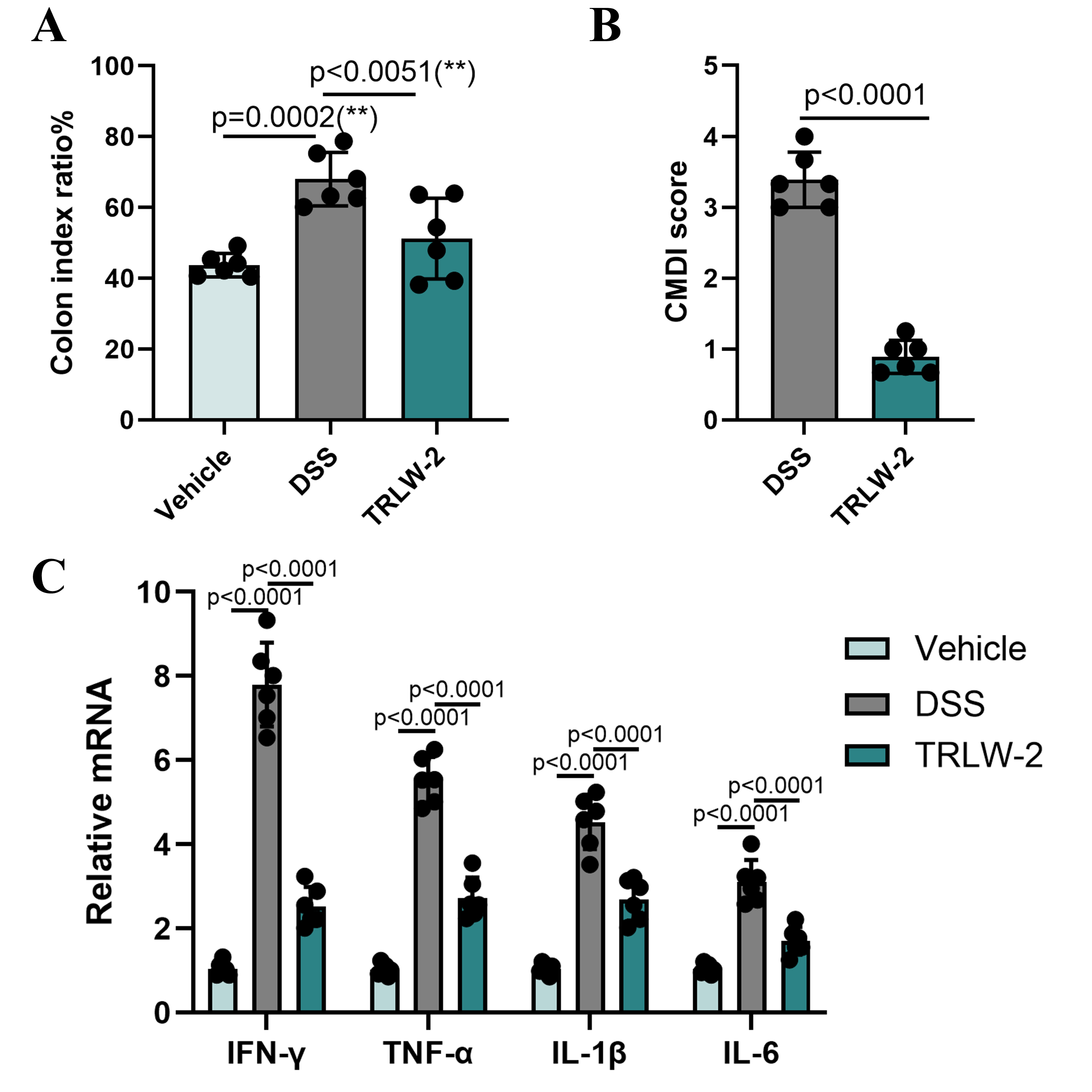

Supplement: Supplementary file 2 [file Image4.tif]

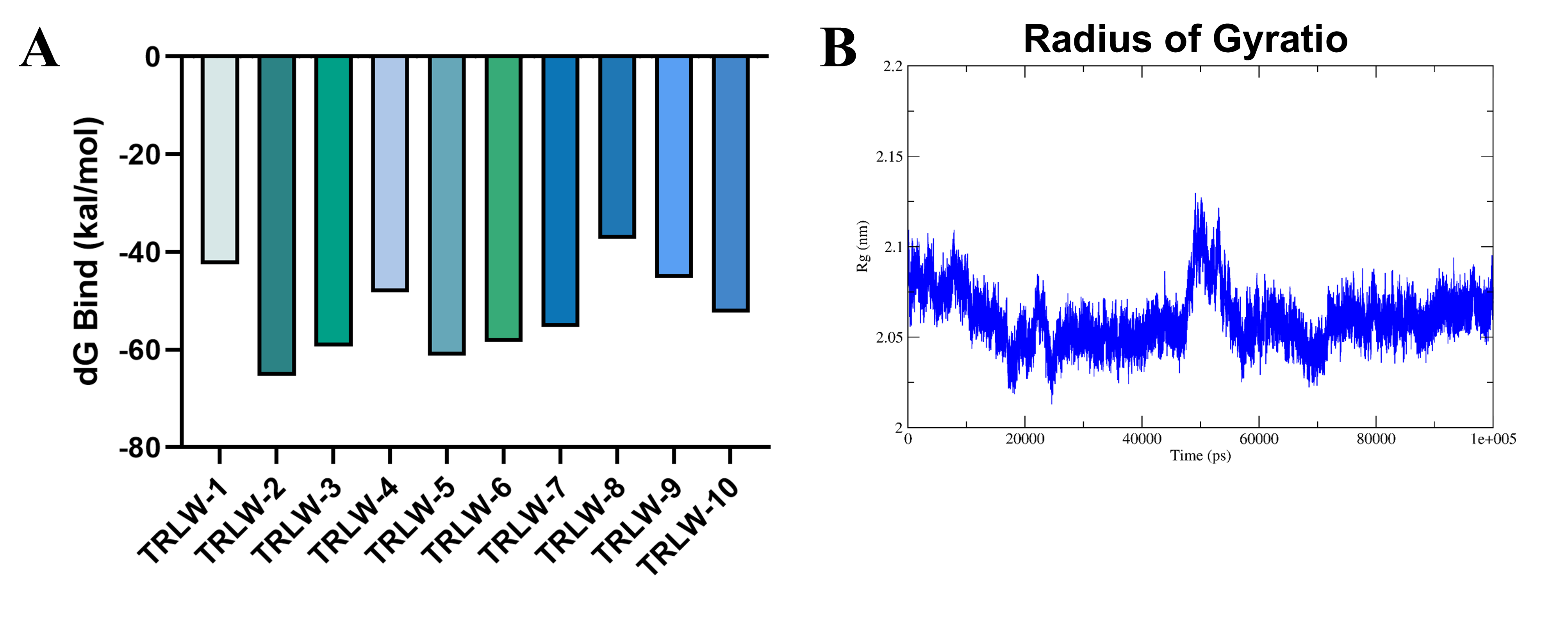

Supplement: Supplementary file 3 [file Image2.tif]

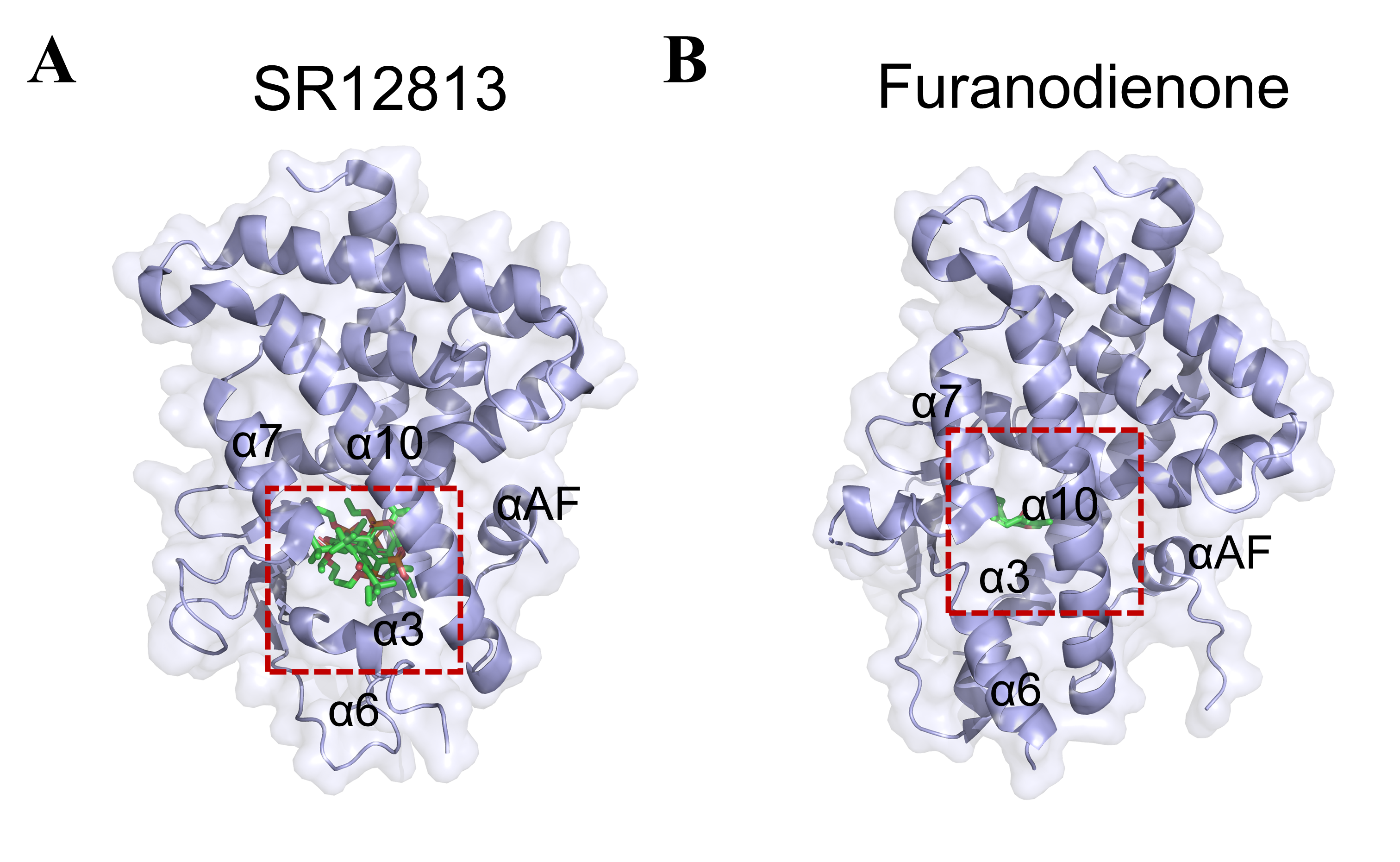

Supplement: Supplementary file 4 [file Image1.tif]
